# Supplementary material for: Differential activity of transcription factors and neuronal effectors during the development of pikeperch brain
Source: Biol Open. 2025 Nov 20;14(11):bio062280. doi: 10.1242/bio.062280 (PMC12673963; doi:10.1242/bio.062280)
Supplement: Supplementary information [file biolopen-14-062280-s1.pdf]

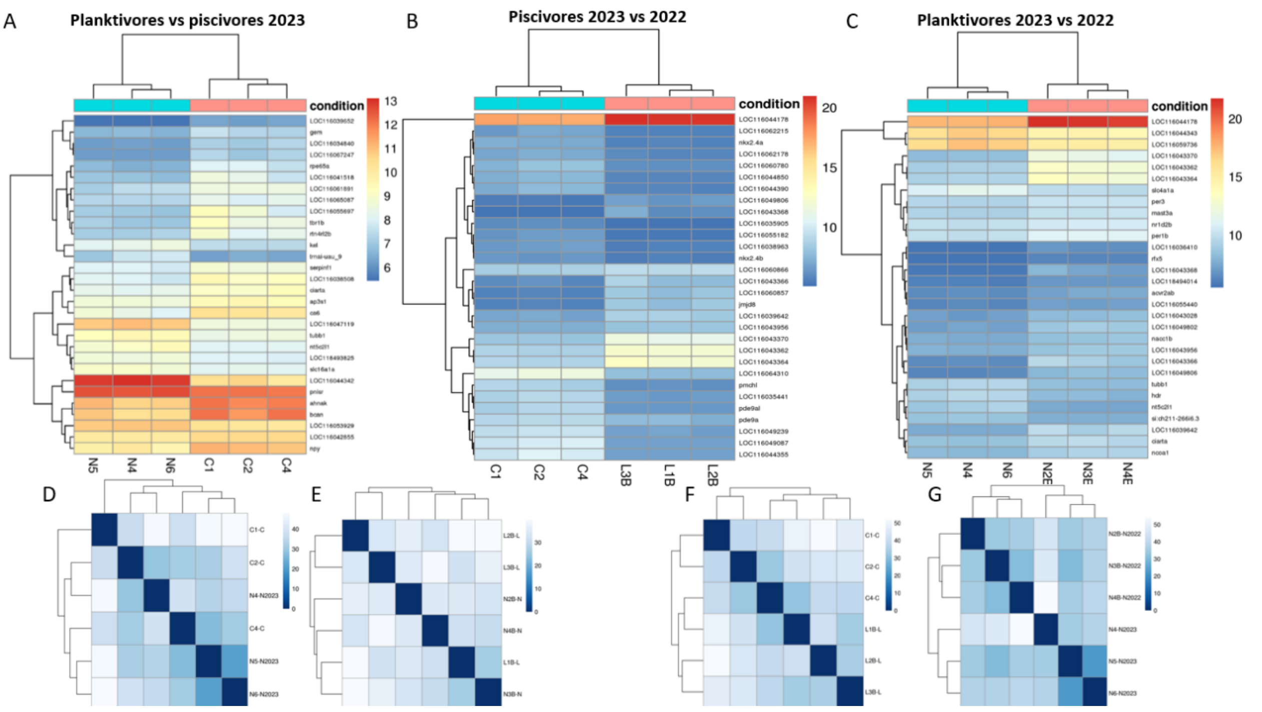

Fig. S1. Sample sequencing statistics. A-C, Bi-clustering heat maps of differentially transcribed genes between growth phenotypes and inter-annual comparisons. D-E, Sample distances show the distances of expression values from each sample. The shorter the distance, the more closely related the samples are. This method is used to identify if the two groups are closely related or not.

Biology Open • Supplementary information

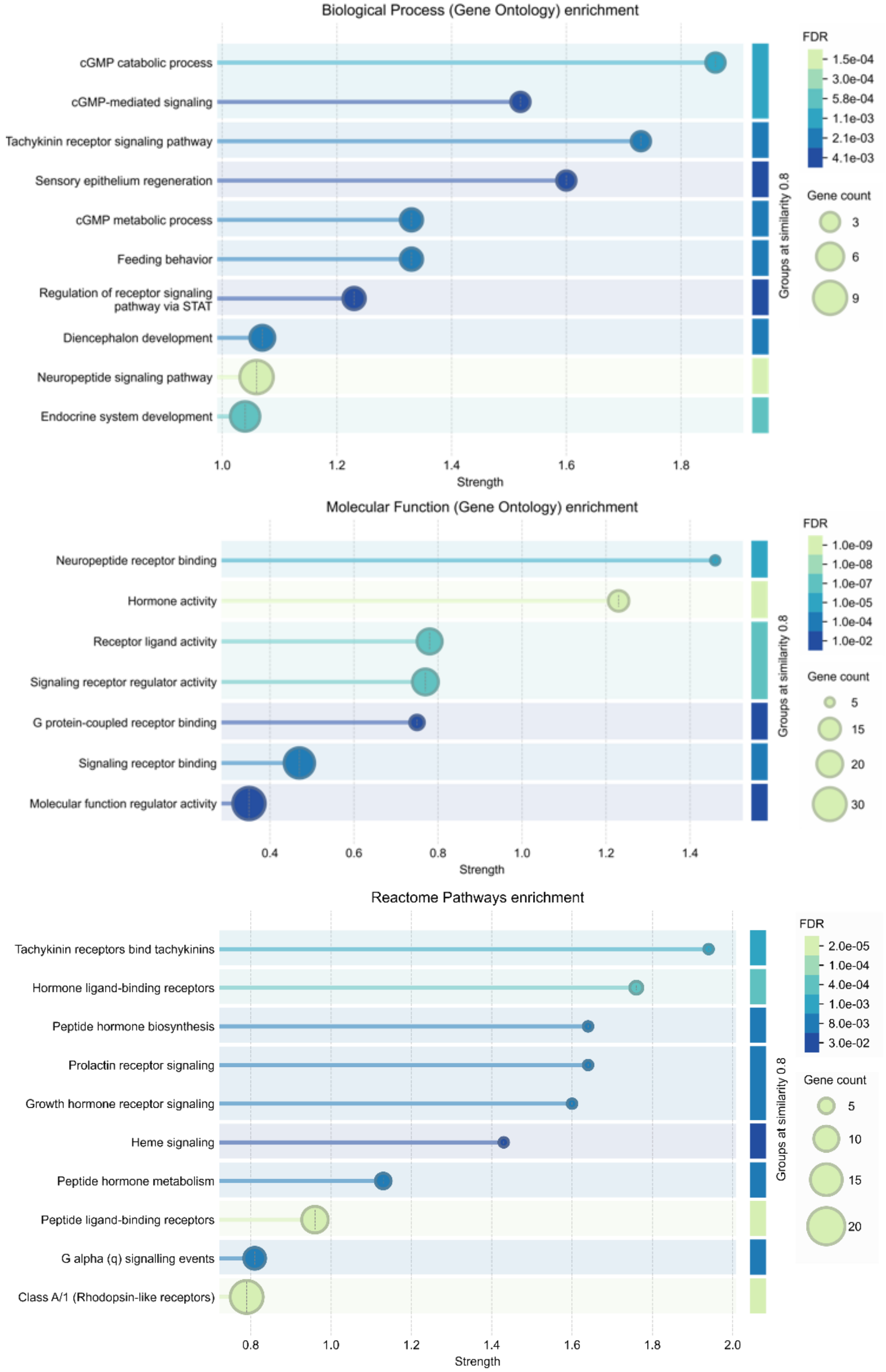

Fig. S2. Inter-annual comparison of GO terms enrichment between years within piscivores. GO terms enriched in the smaller piscivores of 2023 shown, no GO terms were enriched in the larger piscivores of 2022 in comparison to 2023. A single CC GO term was enriched in 2023: ECR; following Annotated Keywords were enriched in 2023: Hormone, Homeobox, Secreted, Signal.

Biology Open • Supplementary information

Biology Open (2025): doi:10.1242/bio.062280: Supplementary information

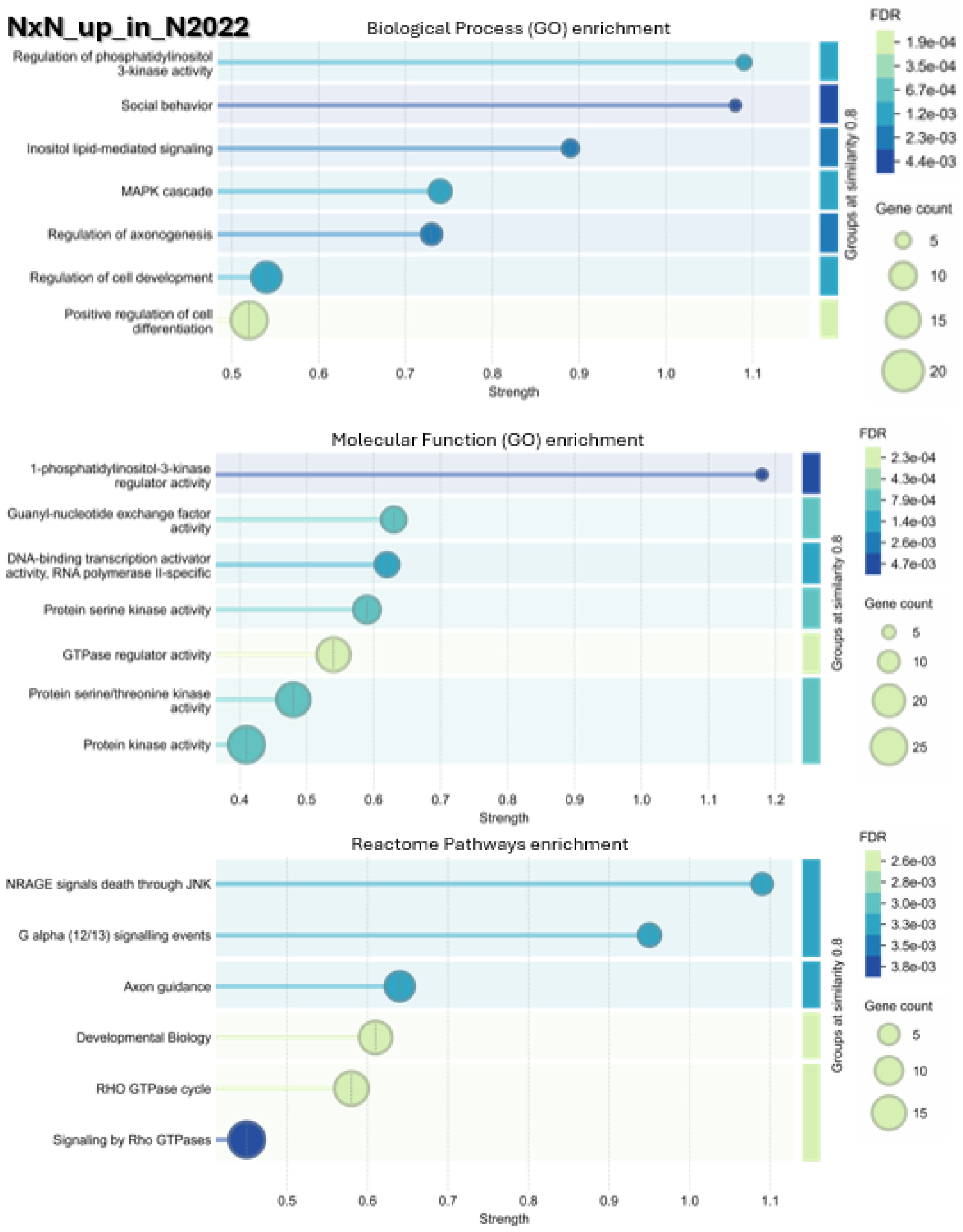

Fig. S3. Inter-annual comparison of GO terms enrichment between years within planktivores. GO term enriched in larger planktivores of 2022 with Reactome Pathways enriched. A single CC GO term was enriched in 2022: Cellular anatomical entity with strength 0.01. A single KEGG Pathway term was enriched in 2022: Phosphatidylinositol signaling system (strength 0.81).

Biology Open • Supplementary information

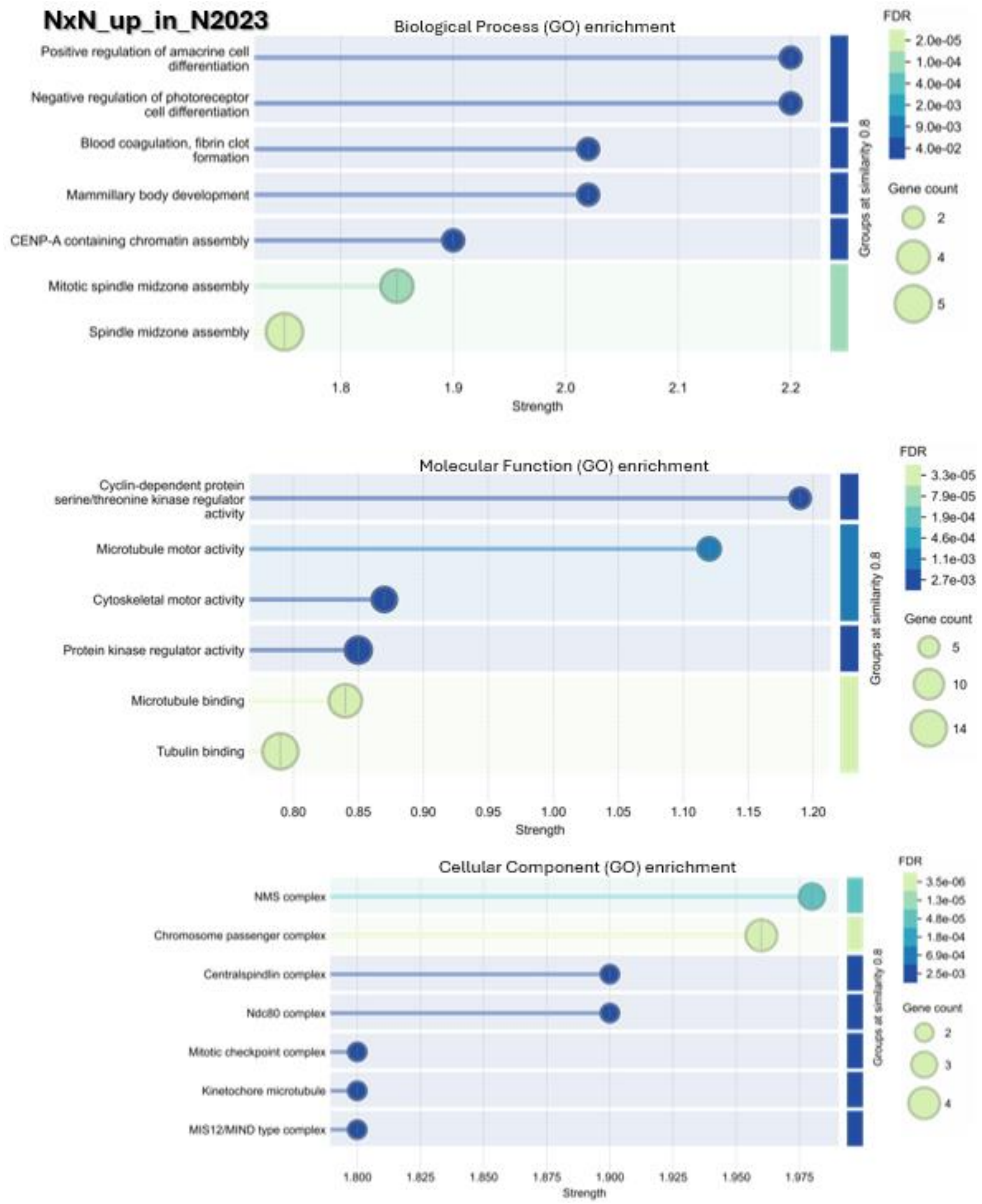

Fig. S4. Inter-annual comparison of GO terms enrichment between years within piscivores. GO term enriched in smaller planktivores of 2023 in comparison with larger planktivores of 2022.

Biology Open • Supplementary information

Table S1. Sample sequencing statistics. Statistics of mapping the reads to the reference genome of pikeperch and quality scores.

Year	Sample ID	Total Reads	Total Mapped	% Total Mapped	Unique Mapped	Yield (Mbp)	Mean Phred Score	% Bases >= 30	RQN
2022	L1B/C1	22,474,046	22,138,772	98.51	18,249,761	6,960	35.2	90.01	10
	L2B/C2	28,257,099	27,811,236	98.42	23,033,036	8,734	35.2	90.1	9.7
	L3B/C3	30,891,143	30,485,550	98.69	23,259,224	9,492	35.47	91.48	10
	N2B	40,188,872	39,635,077	98.62	28,855,296	12,339	35.47	91.44	9.3
	N3B	49,720,977	49,059,287	98.67	37,631,296	15,310	35.41	91.14	10
	N4B	47,858,545	47,149,302	98.52	38,076,666	14,689	35.47	91.43	8.6
2023	C1	41,506,565	40,458,169	98.47	39,409,434	12,934	34.84	88	9.1
	C2	18,801,468	18,345,509	98.57	17,899,377	5,840	34.89	88.13	10
	C4	39,314,275	38,377,177	98.62	37,493,126	12,377	34.67	87.18	10
	N4	29,205,965	28,422,530	98.32	27,681,531	9,240	34.53	86.51	10
	N5	41,290,954	40,261,143	98.51	39,411,070	12,897	34.72	87.37	10
	N6	34,526,267	33,616,414	98.36	32,828,959	10,785	34.78	87.74	10

Explanations
In 2022, two types of tissue were tested for RNA extraction: B - brain, G - gill, but gill did not work in the end

C = Cannibals living in littoral (L), C = L = **piscivores**

N = Normal **planktivorous** fingerlings = **planktivores**

Table S2. Intra annual comparison of differentially transcribed genes between phenotypes in 2023.

Available for download at
https://journals.biologists.com/bio/article-lookup/doi/10.1242/bio.062280#supplementary-data

Biology Open • Supplementary information

Table S3. Inter-annual comparison of differentially transcribed genes in planktivores between 2022 and 2023.

Available for download at
https://journals.biologists.com/bio/article-lookup/doi/10.1242/bio.062280#supplementary-data

Table S4. Inter-annual comparison of differentially transcribed genes in piscivores between 2022 and 2023.

Available for download at
https://journals.biologists.com/bio/article-lookup/doi/10.1242/bio.062280#supplementary-data

Table S5. TFs transcribed in brain of both juvenile pikeperch phenotypes in 2022-2023.

Available for download at
https://journals.biologists.com/bio/article-lookup/doi/10.1242/bio.062280#supplementary-data

Table S6. Slc genes transcribed in brain of both juvenile pikeperch phenotypes in 2022-2023.

Available for download at
https://journals.biologists.com/bio/article-lookup/doi/10.1242/bio.062280#supplementary-data
